# Discovery of peptide probes to modulate oxytocin-type receptors of insects

**DOI:** 10.1038/s41598-018-28380-3

**Published:** 2018-07-03

**Authors:** Peter Keov, Zita Liutkevičiūtė, Roland Hellinger, Richard J. Clark, Christian W. Gruber

**Affiliations:** 1 School of Biomedical Sciences, Faculty of Medicine, The University of Queensland, QLD 4072 Brisbane, Australia; 2 Center for Physiology and Pharmacology, Medical University of Vienna, Schwarzspanierstrasse 17, 1090 Vienna, Austria; 3 Present Address: Victor Chang Cardiac Research Institute, Molecular Pharmacology Division, Lowy Packer Building, 405 Liverpool St, NSW 2010 Darlinghurst, Australia

## Abstract

The oxytocin/vasopressin signalling system is conserved across the animal kingdom. In insects, the role of oxytocin-type (inotocin) neuropeptides has only been studied in locusts, beetles and ants, but their physiology continues to be poorly understood. One reason for this knowledge deficit is the lack of available research tools to complement functional genomics efforts. Consequently, ligands to probe insect inotocin receptors are essential. In this study, we sought to identify novel agonists and antagonists of the inotocin receptor from the representative model species *Tribolium castaneum* and *Lasius niger*. Drawing upon known ligands of the human receptors, we examined the pharmacology of the plant-derived cyclotide kalata B7 and the synthetic oxytocin analogue atosiban. Kalata B7 is a weak partial agonist of both inotocin receptors. This is the first reported direct interaction of cyclotides with an insect receptor, an observation that may explain their presumed role in herbivore defence. Furthermore, we discovered atosiban is an antagonist of the *Tribolium* receptor, which may provide a useful probe to investigate the functionality of inotocin signalling in beetles and related insect species. Our findings will enable further examination of insect inotocin receptor pharmacology and physiology, and may trigger studies to comprehend the interaction of plant cyclotides and insects.

## Introduction

The oxytocin/vasopressin signalling system, consisting of peptide ligands acting on their cognate G protein-coupled receptors (GPCRs), is found across all vertebrate and many invertebrate species^1,2^. In vertebrates, this peptidergic signalling system mediates a range of important physiological functions, with roles spanning the cardiovascular, reproductive, renal and central nervous systems^3,4^. Despite the lack of extensive studies, orthologous invertebrate systems have shown similar physiological functions, including egg-laying behaviours, vascular and gut contractility, and learning behaviours^1,2,5^. However, little is known regarding the role of this system in insect species.

Insects comprise one of the most diverse classes of invertebrate animals. Recent studies suggest that many species within the class Insecta possess an oxytocin-/vasopressin-type peptide system, comprised of a single peptide-receptor pair^6^. The homology of the inotocin peptide system(s) with the oxytocin-/vasopressin-type systems of vertebrates offers unique avenues for studying this conserved peptide-receptor family across evolution.

The first insect oxytocin/vasopressin-type peptide (inotocin: CLITNCPRG-amide) was discovered in the mid-1980s in locusts^7^, and in the meantime, it was found *inter alia* in beetles^8,9^ and ants^10–12^. We have recently explored the oxytocin-type signalling system of the black garden ant, *Lasius niger*, and identified a novel selective antagonist of the human V_1a_ vasopressin receptor^11^. Consequently, efforts to study the biology of insect oxytocin-type peptide signalling will provide greater opportunities towards understanding this peptide-receptor family and realising potential innovations for drug design applications.

One of the best studied insect species with respect to oxytocin-/vasopressin-type signalling to-date (fruit flies and honey bees notably lack this signalling system), is the red flour beetle, *Tribolium castaneum*^8,9^. Being a common pest species, *T. castaneum* represents an essential model organism for basic and applied research, offering discoveries for both medical and agro-economic importance. However, since the initial identification of the inotocin peptide and its cognate receptor in *T. castaneum*^8,9^, little more has been done to examine this system in insects. Amongst other reasons, this may be attributed to a paucity of known pharmacological probes for inotocin receptors.

This provided the rationale to identify new peptide ligands for inotocin receptors. Based on the evolutionary homology of the inotocin system with the human oxytocin/vasopressin system, we explored the pharmacological activity of non-animal-derived peptide ligands. Using a heterologous cell system, we exemplarily examined the functional activity of two known oxytocin/vasopressin receptor ligands, the plant-derived cyclic peptide kalata B7^13^ and the clinically used synthetic oxytocin/vasopressin antagonist atosiban. We examined the effect of these peptides at the inotocin receptor from the insect model species *T. castaneum* and used for comparison the related inotocin receptor of the black garden ant, *Lasius niger*.

## Materials and Methods

### Reagents

Dulbecco’s Modified Eagle’s Medium (DMEM), penicillin/streptomycin and Lipofectamine 2000® were sourced from Thermo Fisher Scientific (Scoresby, Australia). Foetal Bovine Serum (FBS) was sourced from GE Life Sciences (Parramatta, Australia). The IP-One assay kit was purchased from CisBio (Codolet, France). Atosiban was purchased from Sigma Aldrich (Merck; Sydney, Australia).

### Peptide synthesis

**Figure 1:**
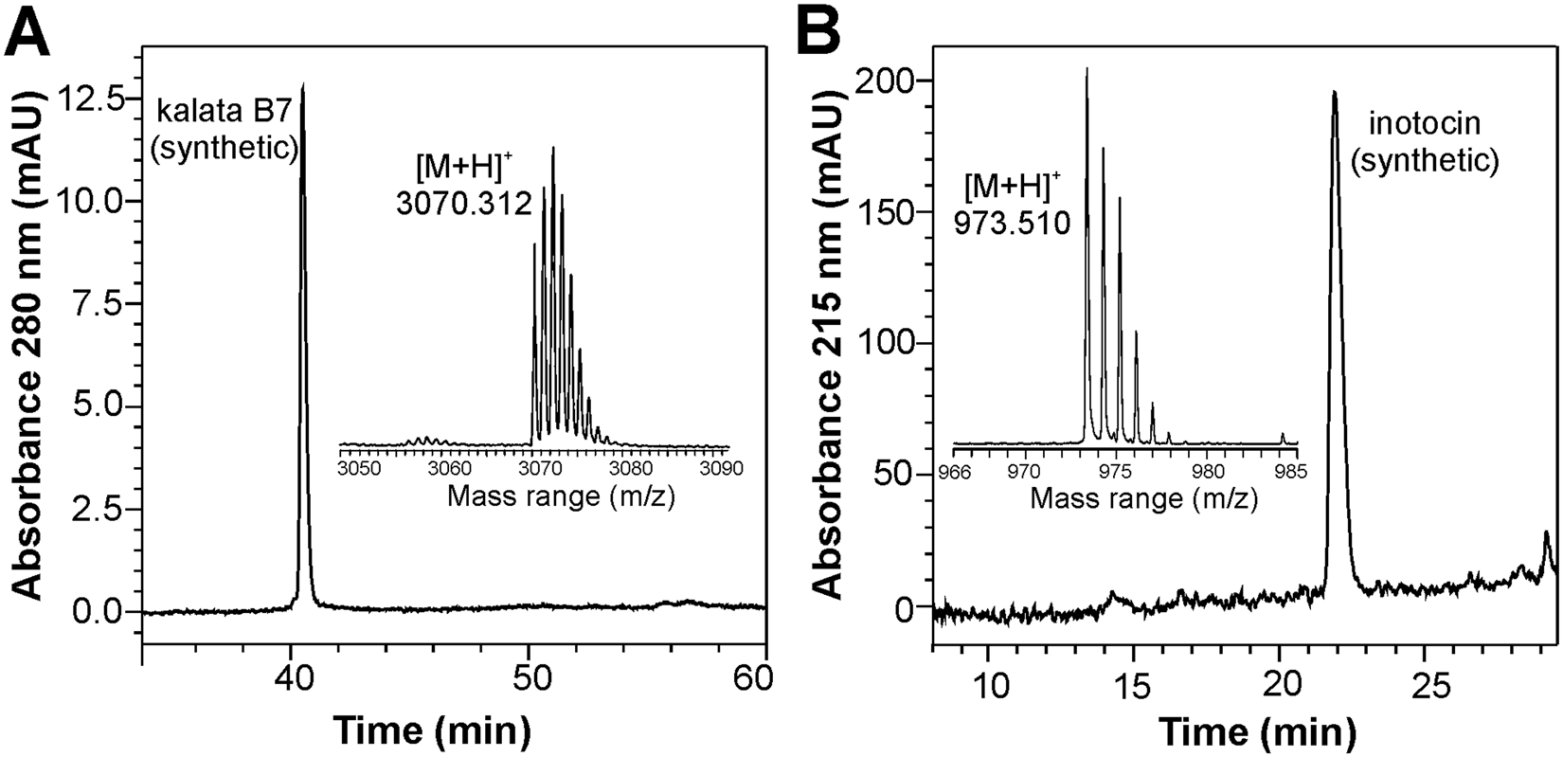
Quality control of synthesised peptides. (**A**) kalata B7 (molecular weight, MW = 3069.27 Da) and (**B**) inotocin (MW = 972.46 Da) were analysed with RP-HPLC and MALDI-TOF mass spectrometry. The A_280_ chromatogram for kalata B7 indicated a purity >95% and the obtained monoisotopic mass signal (3070.3 m/z) corresponds to the oxidized cyclic form of the peptide (insert). The A_215_ chromatogram for inotocin indicates a purity >95% and a monoisotopic mass signal of 973.5 m/z is labelled in the insert.

Inotocin and kalata B7 were synthesised using Fmoc-solid-phase peptide synthesis, where peptide cyclisation was performed via native chemical ligation^11,13–15^. After cleavage from resin, peptides were oxidized in 0.1 M ammonium bicarbonate at pH 8.2 for 24 h. Peptides were purified on reversed-phase high performance liquid chromatography (HPLC) to yield >95% purity and assessed by matrix-assisted laser desorption ionisation time-of-flight (MALDI-TOF) mass spectrometry (Fig. 1). HPLC was carried out according to published protocols^11,13^. Mass spectra were recorded on an autoflex MALDI-TOF instrument (Bruker Daltonics, Bremen). The mass accuracy was calibrated on a daily basis according the manufacturers’ advice using Peptide calibration standard II ranging from ~700–3200 Da (Bruker). The mass spectrometer was operated in reflector mode and instrument settings, such as laser intensity or digitizer gain, were set to produce optimal signal-to-noise ratio and signal intensity in the mass range of 1000–4000 m/z. For sample preparation 3 µL of α-cyano-4-hydroxy cinnamic acid matrix (saturated in acetonitrile/H_2_O/TFA, 50:50:0.1, v/v/v) was mixed with 0.5 µL of peptide solution in aqueous 0.1% TFA. The mixed sample (0.5 µL) was transferred on a standard 384 MTP target plate and allowed to air dry in the dark. Data processing was performed with Compass v1.7 and flexAnalysis v3.4 (Bruker).

### cDNA constructs and transfections

The *T. castaneum* receptor gene was kindly provided by Yoonseong Park (Kansas State University)^8^, whereas the *L. niger* inotocin receptor gene had been cloned and sequenced as per Di Giglio *et al*.^11^. Both receptor constructs were subsequently cloned into the pEGFP-N1 expression plasmid, generating C-terminal EGFP fusion receptor proteins. Receptors were expressed in HEK293 cells (ATCC, CRL 1573) via transfection using the Lipofectamine 2000® transfection reagent. The use of mammalian cell lines to study the receptors of insects and other invertebrate species, in the same vain as orphan receptors from mammalian genomes, is considered a standard procedure. Various studies have utilized this approach^8,9,11^. Whilst such mammalian cell lines may not completely mimic insect cell biology, GPCRs and their signalling is highly homologous across the animal kingdom; it would be unlikely that the insect receptors would mis-fold in a mammalian cell line. Nevertheless, during assay optimization we routinely monitored surface expression of GFP-tagged receptors, and we only proceeded to pharmacological assays, if the receptor localized at the cell membrane. Moreover, most biochemical cell-based functional assays are optimized for mammalian cells. Whilst it is possible that insect cells could be utilized for these assays, it is unknown whether they endogenously express inotocin receptors. Hence, the use of a mammalian cell system facilitates a cell background devoid of endogenous insect receptors. Accordingly, mammalian cells were cultured in DMEM supplemented with 10% FBS and penicillin/streptomycin (100 U mL^−1^) and plated in 6-well plates prior to transfection. When cells had reached confluency, cells were transfected as per the manufacturer’s protocol with 2.5 µg per well of each respective receptor construct using a DNA:Lipofectamine 2000® ratio of 1:3 prepared in serum-free DMEM. After 4–6 h, cells were dissociated by trituration and replated into white opaque 384-well plates (CellStar®, Greiner, Kremsmünster, Austria) at approximately 10,000 cells per well. Cells were cultured for approximately 38–48 h in a humidified incubator at 37 °C and an atmosphere of 5% CO_2_, and then assayed for second messenger production. Verification of receptor expression was assessed via microscopic examination of membrane localised GFP fluorescence.

### Measurement of inositol phosphate accumulation

Quantitative measurements of receptor-mediated inositol 1-phosphate (IP_1_) were performed by competitive immunoassay utilising the IP-One assay kit (CisBio, France). HEK293 cells were prepared as described above and then assayed as per the manufacturer’s protocol. Briefly, at the time of assay, the culture media was removed from cells, and then replaced with IP-One stimulation buffer (HEPES 10 mM, CaCl_2_ 1 mM, MgCl_2_ 0.5 mM, KCl 4.2 mM, NaCl 146 mM, glucose 5.5 mM, LiCl 50 mM, pH 7.4). Cells were allowed to equilibrate in the stimulation buffer at 37 °C for 15 min, followed by addition of peptide ligands (to final concentrations of 0.1 pM – 10 µM) for subsequent stimulation for 1 h at 37 °C. The stimulation was terminated by lysis and the simultaneous addition of homogenous time-resolved fluorescence resonance energy transfer reagents. The lysates were incubated for a minimum of 1 h at room temperature. Fluorescence emission measurements at 620 nm and 665 nm were performed using a FlexStation3 plate reader (Molecular Devices, Sunnyvale, CA) or Spark Multimode plate reader (Tecan, Männedorf, Switzerland) at an excitation wavelength of 340 nm. Results were analysed as a ratio of fluorescence intensities of 665 nm to 620 nm.

### Data analysis

Data from cellular assays were analysed using GraphPad Prism 7.03 (GraphPad Software, San Diego). Concentration-response data were fitted to three- or four-parameter logistic equations to derive estimates of potency (LogEC_50_) and efficacy (E_max_), relative to the maximal inotocin control response. Data from agonist-antagonist interaction experiments were fit to a Schild regression analysis^16^.

### Data availability

The datasets generated during and/or analysed during the current study are available from the corresponding author on reasonable request.

## Results and Discussion

The inotocin peptide signalling system of insects presents a variety of opportunities for biology. Exploitation of their evolutionary homology and phylogenetic distribution, offers advances in insect biology and applications for drug design^6,11^. Herein, we have described the pharmacological characterisation of two oxytocin/vasopressin peptide analogues, kalata B7, a plant-derived cyclic peptide, and atosiban, a synthetic, clinically used oxytocin/V_1a_-receptor antagonist. These findings will have implications to understand the function of oxytocin-/vasopressin-type signalling across the arthropods.

### Identification of a plant-derived peptide agonist of insect oxytocin/vasopressin-type receptors

**Figure 2:**
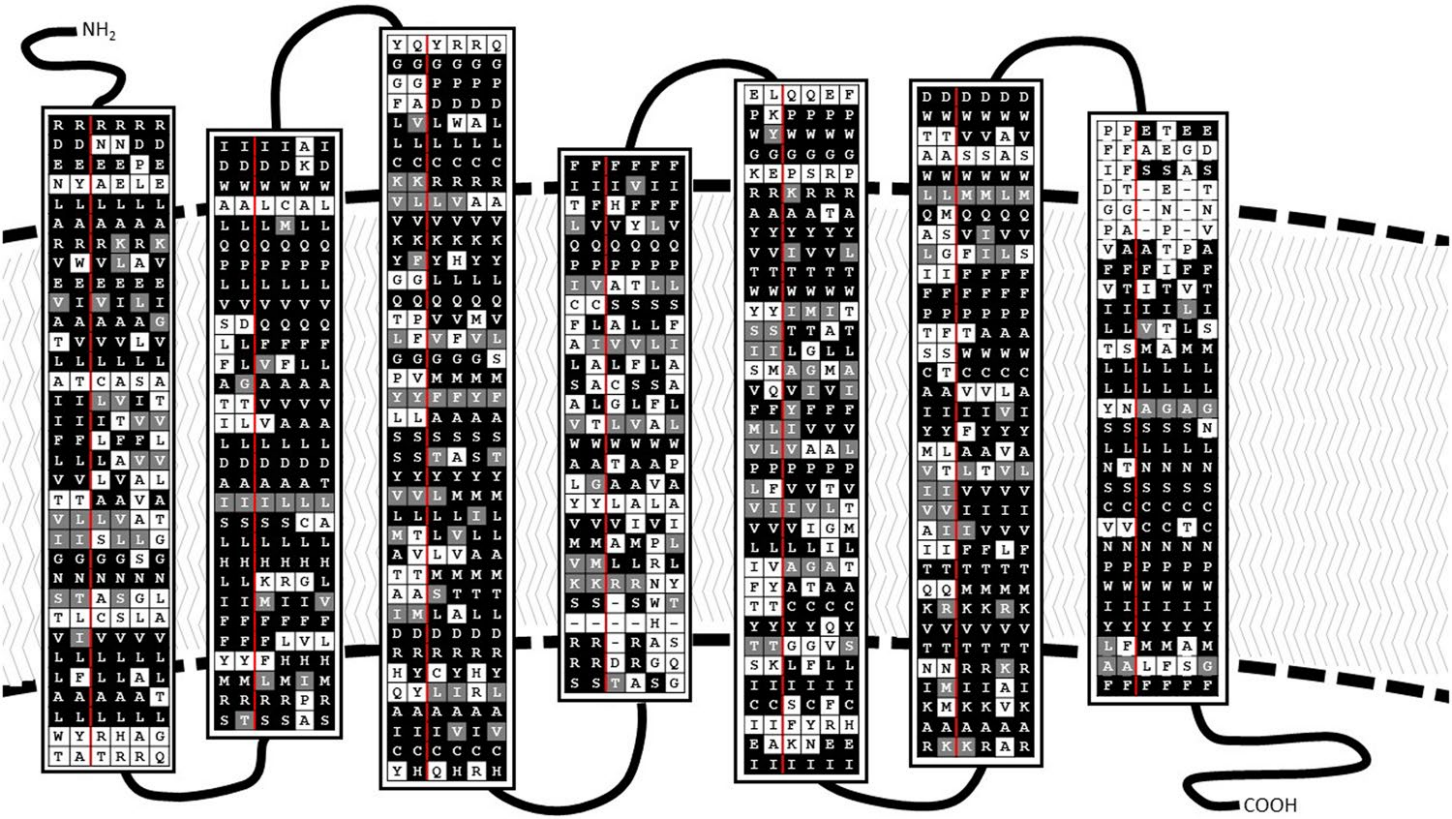
Comparison of insect inotocin and human oxytocin/vasopressin receptor sequences in transmembrane helical domains. Amino acid sequence alignments, performed with Clustal Omega, of the transmembrane helices of each receptor are shown vertically, with homologous regions highlighted in Boxshade representation. Receptor sequences are, from left to right, *T. castaneum* inotocin receptor, *L. niger* inotocin receptor (separated with a red line), human oxytocin receptor, human vasopressin V_1a_ receptor, human vasopressin V_2_ receptor and human vasopressin V_1b_ receptor.

**Figure 3:**
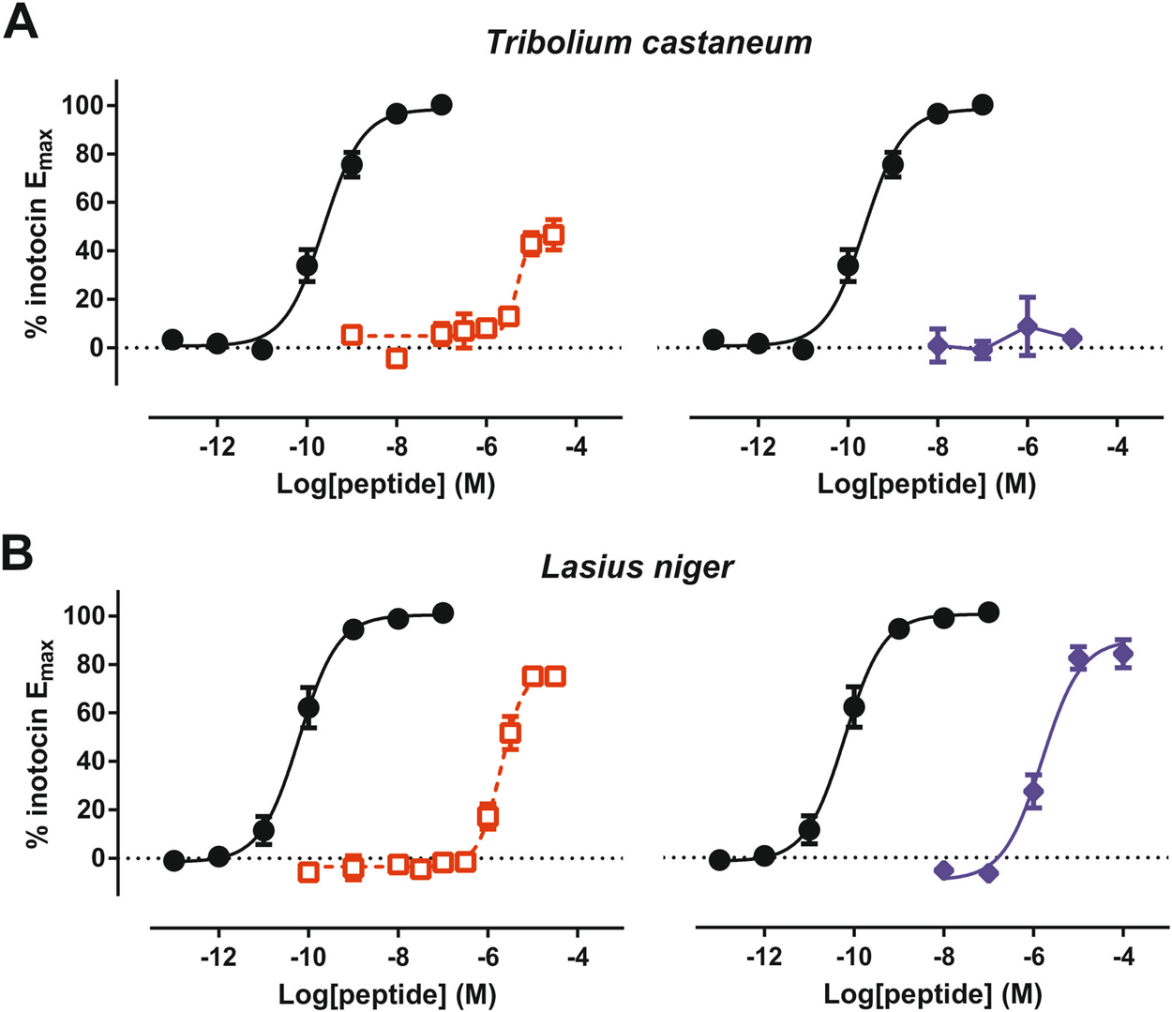
Concentration-dependent inositol phosphate IP_1_ accumulation in HEK293 cells expressing inotocin receptors of (**A**) *T. castaneum* and (**B**) *L. niger*. Concentration-response curves were constructed by challenge of receptor-expressing cells to inotocin (solid circles, solid line), kalata B7 (open red squares, dashed line), or atosiban (solid blue diamonds, solid line), at final concentrations ranging from 0.1 pM to 100 µM. Data are presented as mean ± S.E.M. of at least three independent experiments conducted in triplicate. For clarity we are presenting the curves of kalata B7 (left) and atosiban (right) in separate panels, each with the same endogenous control dataset.

Previous investigation into the ethnopharmacological use and uterotonic activity of herbal extracts from *Oldenlandia affinis* identified the cyclic peptide, kalata B7, as a partial agonist of human vasopressin V_1a_ and oxytocin receptors^13^. Scrutiny of the amino acid sequences of the insect and human oxytocin/vasopressin(-type) receptors (Table 1), particularly of the transmembrane helices (Fig. 2), reveals considerable homology between each of the receptors. Compared to the level of identity between the *T. castaneum* inotocin receptor and its human counterparts (approximately 37–40% similarity), the human vasopressin V_1a_ receptor exhibits similar levels of identity compared to the other human receptor subtypes (approximately 38–40% similarity). Further focus on the homology of the transmembrane helices and extracellular loops of the receptors, where the peptide binding site presumably is localised, reveals approximately 46–50% similarity between the *T. castaneum* inotocin receptor and its human counterparts. Similar comparative analysis of this region from each of the human receptor subtypes found 49–64% identity between the human proteins. Given this homology between human oxytocin/vasopressin receptors and insect inotocin receptors, we determined whether the cyclotide kalata B7 can modulate inotocin receptor function. Examination of receptor-mediated IP_1_ accumulation after incubation with the plant peptide determined that kalata B7 was a weak partial agonist of both inotocin receptors (Fig. 3; Table 2). Compared to the sub-nanomolar potency of the endogenous inotocin peptide at the *T. castaneum* and *L. niger* inotocin receptors (approx. 230 pM and 62 pM, respectively), kalata B7 exhibited low micromolar potency (7.2 µM and 2.2 µM, respectively).

**Table 1:** Homology between insect inotocin receptors and human oxytocin/vasopressin receptors.

	*Tribolium castaneum* inotocin receptor (% identity)	*Lasius niger* inotocin receptor (% identity)
*Tribolium castaneum/Lasius niger* inotocin receptors	53.1 (62)*	
human oxytocin receptor	40.4 (49)	36.1 (44)
human V_1a_ vasopressin receptor	38.6 (50)	36.4 (45)
human V_2_ vasopressin receptor	37.8 (48)	35.7 (44)
human V_1b_ vasopressin receptor	37.0 (46)	34.2 (42)

*Values in parentheses correspond to percentage of amino acid identity of receptor sequences following exclusion of the N-terminal, C-terminal and intracellular loop domains.

**Table 2:** Potency and relative efficacy of kalata B7 and atosiban at the *T. castaneum* and *L. niger* inotocin receptors.

	**LogEC**_**50**_ (Log[M])	**E**_**max**_ (% inotocin max. response)
***Tribolium castaneum***		
inotocin	−9.63 ± 0.08	100
kalata B7	−5.14 ± 0.22	60.3 ± 9.6
atosiban	*n.d*.	*n.d*.
***Lasius niger***		
inotocin	−10.21 ± 0.08	100
kalata B7	−5.66 ± 0.10	86.5 ± 4.7
atosiban	−5.81 ± 0.12	89.9 ± 4.5

Data are presented as mean ± S.E.M. from at least three independent experiments, *n.d*. not determined.

We hypothesised that kalata B7 would engage inotocin receptors based on the homology of the insect receptors with their human counterparts. Although cyclotides have been reported to exhibit insecticidal activities^17–21^, this is to our knowledge the first time a cyclotide, i.e. kalata B7, has been demonstrated to directly interact with an insect protein target. Preliminary characterisation of the *T. castaneum* inotocin receptor suggests that activation of the inotocin signalling cascade is unlikely to mediate insecticidal activity via cytotoxic mechanisms^8^. Indeed, we did not observe negative effects on cell viability at the concentrations tested (data not shown), consistent with the reported high micromolar haemolytic potency of cyclotides^22,23^. Consequently, together with the characterisation of low micromolar potency of kalata B7 at both the *T. castaneum* and *L. niger* receptors, it appears unlikely that these receptor-mediated actions would cause direct insecticidal or membrane disruptive actions. Rather, interaction of the cyclotide with inotocin receptors could modulate physiology or behaviour of the insects.

While a detailed mechanism of inotocin signalling and its physiology are not entirely known, the expression and localisation profiles of inotocin and its receptor in *T. castaneum* suggest a role in development and/or as neuromodulator, with few or no direct peripheral physiological actions^8,9^. Recent work by Chérasse and Aron^10^ also suggest similar neurophysiological and/or developmental roles of inotocin in *L. niger*. It is beyond the scope of this study to explore the nature and extent of such *in vivo* effects, but chronic modulation of inotocin receptors may have negative effects on insect reproduction and/or development. Hence the observed pharmacological interaction of cyclotides with inotocin receptors could indirectly control insect survival. We understand this is a hypothesis to be explored in future studies, but the notion that some plants produce cyclotides to control the population of insects that feed on them could be one possible explanation for the evolution of cyclotides, namely to fend-off herbivorous insects.

An interaction between cyclotide-expressing plants and insects is plausible. Cyclotides are estimated to be produced to levels of up to 1 g per kg of wet plant weight^23^. Given the chemical and enzymatic stability of cyclotides, the ingestion of microgram quantities of plant material would be sufficient for delivery/accumulation of cyclotide to micromolar concentrations in an individual insect. Such concentrations would be adequate for a cyclotide, such as kalata B7, to engage with inotocin receptors. Whether cyclotide-inotocin receptor interactions natively occur remains to be elucidated. Nonetheless, the oral delivery of cyclotides with distinct GPCR-modulating activities may prove valuable for the design of peptide-based pharmacological probes in insects, which could be used to study the *in vivo* functionality of inotocin signalling or be developed for agricultural applications. For instance, engineering of kalata B7 or other cyclotides to yield more potent peptide ligands targeting inotocin receptors could yield peptides to affect insect survival and control insect populations. Importantly one should bear in mind that cyclotides, for instance by application as insecticidal spray or expressed in genetically-modified plants for herbivore defence, may potentially target peptide receptors of pest, as well as non-pest insects, and thereby may modulate the physiology and survival of ‘good and bad’ insect species.

### Identification of an inotocin receptor antagonist from a synthetic oxytocin analogue

**Figure 4:**
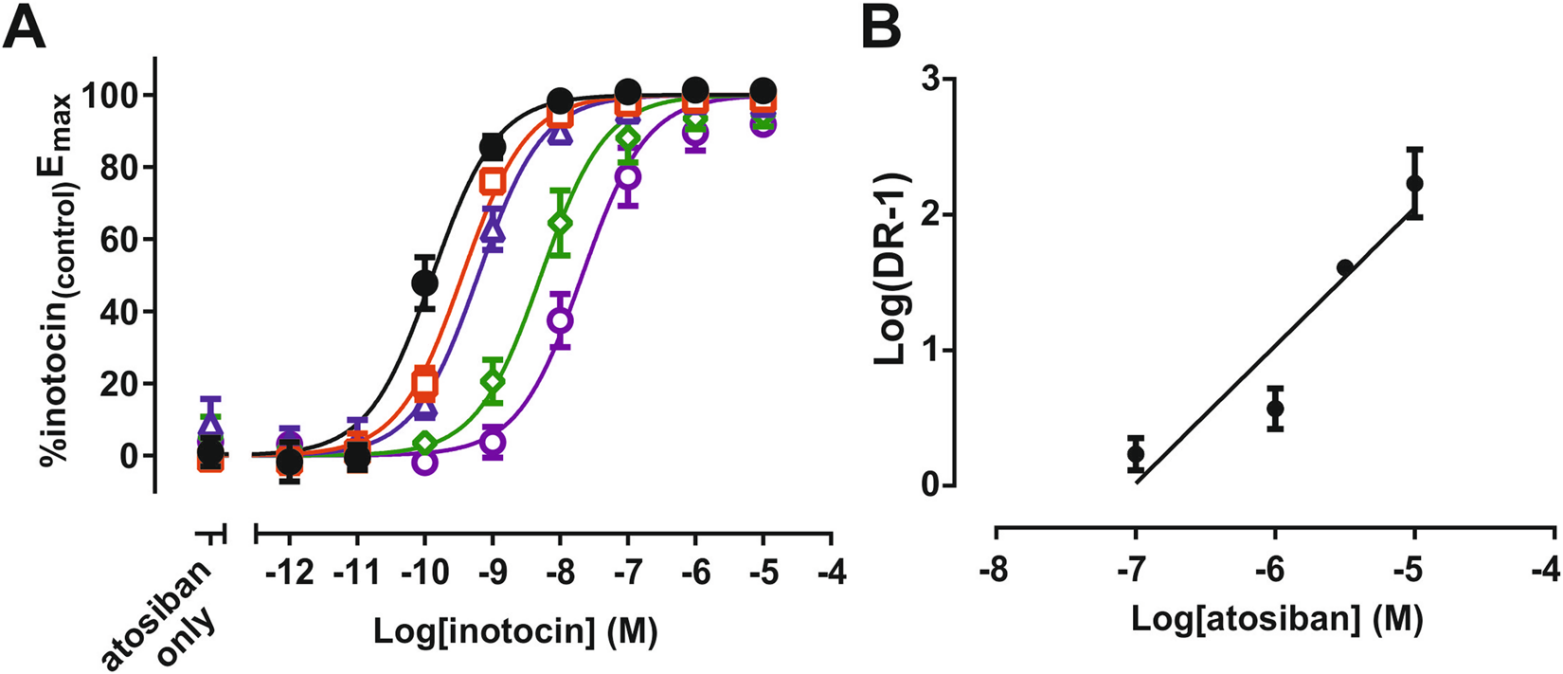
Atosiban competitively antagonises inotocin-mediated IP_1_ accumulation in HEK293 cells expressing the *T. castaneum* inotocin receptor. (**A**) Concentration-dependent rightward displacement of the concentration response curve by atosiban. Cells were stimulated with varying concentrations of inotocin (1 pM – 10 µM, filled black circles) in the absence or presence of 100 nM (open red squares), 1 µM (open blue triangles), 3 µM (open green diamonds), and 10 µM (open purple circles) atosiban. (**B**) Schild analysis of antagonist shifts. Data are presented as mean ± S.E.M. of three independent experiments conducted in triplicate. pA_2_ = 7.03 ± 0.086, Schild Slope = 1.00 ± 0.085.

In parallel to characterising the inotocin receptor activity of kalata B7, we sought to identify an antagonist peptide. Our recent study with the *L. niger* inotocin peptide-receptor system^11^ further emphasised the structural homology shared between inotocin receptors and human oxytocin/vasopressin receptors (Fig. 2, Table 1). In comparison to the human receptors, the *T. castaneum* inotocin receptor (as well as the *L. niger* receptor) exhibits greatest identity with the oxytocin and vasopressin V_1a_ receptor. Given this homology, we were interested in the ability of the oxytocin/V_1a_ receptor antagonist, atosiban, to interact with the two inotocin receptors. Moreover, given that initial characterisation of the *T. castaneum* receptor reported oxytocin as an agonist, but not arginine-vasopressin^8^, we hypothesised that the insect receptors may share functional similarities with the human oxytocin receptor and that atosiban may behave as an antagonist at these receptors. Initial screening found that atosiban lacked agonist activity at the *T. castaneum* inotocin receptor (Fig. 3A), but was a partial agonist at the *L. niger inotocin* receptor (Fig. 3B, Table 2). Subsequent interaction studies of inotocin and atosiban at the *T. castaneum* inotocin receptor found that atosiban was capable of antagonising the inotocin peptide in a concentration-dependent fashion, as demonstrated by dextral displacement of the agonist curve (Fig. 4). Schild analysis of these data indicates atosiban competitively inhibits the *T. castaneum* inotocin receptor with an estimated functional affinity of approximately 93 nM (pA_2_ = 7.03 ± 0.086, Schild Slope = 1.00 ± 0.085).

Despite the evolutionary conservation of the inotocin peptide sequence in both species, it is interesting that atosiban selectively antagonises the *T. castaneum* receptor. These findings support the potential translation of pharmacological activity of a ligand from one species to another^24,25^. Further interrogation will be required to identify the structural differences between the *T. castaneum* and *L. niger* receptors responsible for this activity profile. Nonetheless, atosiban presumably interacts with other insect inotocin receptors, and antagonizes receptors of species closely related to *T. castaneum*. As such, the commercially-available oxytocin/vasopressin receptor antagonist atosiban could prove useful as research tool for inotocin receptors.

This opens avenues to study the physiology of the inotocin system *in vivo*. Preliminary gene knockdown studies have indicated that the inotocin system is not critically involved in the mortality, pupal development, and egg laying and hatching of *T. castaneum*^8^. As outlined above, the predominant expression of this system in the beetle’s central nervous system^8,9^, suggests that the inotocin system may largely play a role in neuro-behavioural roles in *T. castaneum*. Specifically, the authors report that inotocin has an indirect diuretic effect in the beetle (i.e., inotocin does not act on the Malpighian tubules directly), rather for inotocin to cause an increase in urine production, parts of the nervous system needs to be present. Similarly, inotocin signalling is thought to regulate diuresis in locusts^7^. Unlike genetic approaches, pharmacological interference by antagonists, such as atosiban will allow for greater temporal and localised control over disruption of this peptide signalling system *in vivo*. Such studies will determine the utility of the beetle’s – and other model insects – inotocin system as a homologous model for physiology and/or behaviour in other invertebrate species.

### Concluding Remarks

This study details the identification of two new ligands for insect inotocin receptors. These ligands may prove useful as insect receptor probes in the future. With such probes, greater opportunities to study the inotocin peptide receptor system in *T. castaneum* and other insect species are available. Kalata B7’s receptor activity warrants further study into the potential role for cyclotides to target specific insect receptors and proteins, and whether such activities have any biological relevance to their characterised insecticidal actions. Moreover, the ability to antagonise inotocin receptors will facilitate advancements and provide research tools into understanding the role of inotocin and possibly other neuropeptide signalling systems in beetles and related insect species.
